# Effects of underwater birth on the newborn

**DOI:** 10.1590/1516-3180.2014.1323808

**Published:** 2014-04-14

**Authors:** Slobodan Sekulic

**Affiliations:** I MD, PhD. Attending Physician and Senior Associate Scientist, Department of Neurology, University Hospital, Clinical Center of Vojvodina, Novi Sad, Serbia.

In a recently published comment on a systematic review published in the Cochrane Database dealing with immersion in water during labor and birth, the focus was on the benefits for pregnant women. These would include reduction of the need to use epidural/spinal analgesia, reduction of the duration of the first stage of labor, decreased need for episiotomies and higher level of satisfaction with the birth experience.^1^

However, the effects on the newborn relating to underwater birth also need to be taken into consideration. Reports on underwater births have suggested that specific complications may occur in newborns, due to aspiration of water, such as: respiratory distress, blood dilution, hyponatremia, convulsions, hypoxic ischemic encephalopathy and lung infection. Umbilical cord rupture could also occur.^2^ The concept of water birth presupposes that delivering a baby underwater will free the baby from the shock of gravity. Also, water birth should represent a more gentle transition from intrauterine to extrauterine life, and a warm bath serves as an imitation of the weightlessness experienced during the early embryonic stages.^3^ Conditions resembling neutral floating last up to the 20^th^ gestational week and, during this period, the apparent weight of the fetus is 5-10% of its actual weight. During the last trimester of gestation, the apparent weight of the fetus is 60-80% of its actual weight. In other words, the fetus senses more than two-thirds of its actual weight.^4^ Rupture of the amniotic sac during delivery results in complete cessation of the buoyant force on the fetus and gives rise to an apparent increase in body weight of 20-40%. Underwater delivery suddenly exposes the newborn to the effects of buoyant force and puts it back into the conditions of neutral floating after several months of continuously sensing two-thirds of its actual weight. Considering that the fetus starts to have long-term memory from the 34^th^ gestational week onwards,^5^ exposure to neutral floating at birth is the newborn’s first remembered encounter with this physical environment. Underwater birth therefore does not represent a more gentle transition from intrauterine to extrauterine life. Exposure of the newborn to this new environment may induce the orienting reflex, i.e. excitation of the newborn with additional stimulation of the breathing reflex.

It is necessary to carry out randomized controlled trials that would determine whether underwater birth is an exotic mode of delivery that exposes the newborn or the mother to an unnecessary risk or whether it is a delivery mode associated with a reduced risk of complications, compared with classical delivery.
